# Bibliometric Analysis of Antipsychotic-induced Metabolic Disorder from 2006 to 2021 Based on WoSCC Database

**DOI:** 10.2174/1570159X23666241016090634

**Published:** 2024-10-16

**Authors:** Zhihao Guo, Zi Zhang, Lu Li, Ming Zhang, Shanqing Huang, Zezhi Li, Dewei Shang

**Affiliations:** 1 Department of Pharmacy, The Affiliated Brain Hospital of Guangzhou Medical University, 36 Mingxin Road, Guangzhou, China;; 2 School of Pharmacy, Guangzhou Medical University, 1 Xinzao Road, Guangzhou, China;; 3 Key Laboratory of Neurogenetics and Channelopathies of Guangdong Province and the Ministry of Education of China, Guangzhou Medical University, Guangzhou, China;; 4 Department of Nutritional and Metabolic Psychiatry, The Affliated Brain Hospital of Guangzhou Medical University, Guangzhou, China;; 5 Guangdong Engineering Technology Research Center for Translational Medicine of Mental Disorders, Guangzhou, China;; 6 Jiangsu Key Laboratory of Neurodegeneration, Nanjing Medical University, Nanjing, China

**Keywords:** Antipsychotics, metabolic disorder, bibliometrics, visual analysis, LC-MS/MS, homeostasis

## Abstract

**Background:**

With the frequent use of antipsychotics, the metabolic disorder (MetD) caused by drugs has received increasing attention. However, the mechanism of drug-induced MetD is still unclear and is being explored. Keeping abreast of the progress and trending knowledge in this area is conducive to further work.

**Objective:**

The aim of this study is to analyze the latest status and trends of research on antipsychotic-induced metabolic disorder (AIMetD) by bibliometric and visual analysis.

**Methods:**

3478 publications of AIMetD from 2006 to 2021 were retrieved from the Web of Science Core Collection database. R-biblioshiny was used for descriptive analysis, CiteSpace for cooperative network, co-citation analysis and burst detection, and VOSviewer for co-occurrence keywords was used.

**Results:**

Since 2006, the publications have been growing fluctuantly. These studies have extensive cooperation among countries/regions. The most influential country/region, institution and author are the USA, King's College London and Christoph U Correll. Analysis of references shows the largest cluster of “antipsychotic-induced metabolic dysfunction”, which is an important basis for MetD. The recent contents of the burst citation are related to “glucose homeostasis” and “cardiovascular metabolism”. Several bursting keywords were discerned at the forefront, including “LC-MS/MS”, “major depressive disorder”, “expression”, and “homeostasis”.

**Conclusion:**

The AIMetD study is in a state of sustained development. Close cooperation between countries/regions has promoted progress. For grasping the foundation, development, and latest trends of AIMetD, it is recommended to focus on active institutions and authors. Based on AIMetD, subdivision areas such as “LC-MS/MS”, “expression”, and “homeostasis” are forefronts that deserve constant attention.

## 1 INTRODUCTION

Antipsychotics (APs) are commonly used to treat schizophrenia, bipolar disorder and other psychiatric brain diseases [1]. Medications can be divided into typical antipsychotics (TAPs) and atypical antipsychotics (AAPs) according to the induction of extrapyramidal reactions and delayed motor disorders [2]. TAPs, also known as first-generation antipsychotics, primarily act by antagonizing dopamine D_2_ receptors, are effective only for positive symptoms and are prone to severe extrapyramidal reactions [3]. AAPs, also known as second-generation antipsychotics, can relieve the positive, negative and cognitive symptoms of psychosis [4]. AAPs have an affinity for dopamine receptors, 5-hydroxytryptamine (5-HT) receptors, muscarinic cholinergic receptors, noradrenergic receptors, histamine receptors and glutamate receptors *in vivo* and exert their antipsychotic effects mainly by antagonizing D_2_ receptors and 5-HT_2_ receptors [5].

With widespread clinical use and further research, APs have been found to be associated with the development of metabolic disorder (MetD) [6]. The diagnostic criteria for metabolic syndrome (MetS) are that the patient has weight gain and any two of hypertension, abnormal glucose metabolism or abnormal lipid metabolism [7]. Patients treated with APs may not necessarily reach the level of MetS, but the risk of weight gain, diabetes, dyslipidemia and cardiovascular disease increase. Due to being able to relieve the positive, negative and cognitive symptoms and weaker extrapyramidal syndrome, APPs are more commonly used in practice. However, the mechanism of APP-induced metabolic disorder (AIMetD) is not fully understood. Hermida *et al.* hypothesized that it is the result of a combination of exogenous and endogenous factors, including the type and dose of medication, duration of administration, gender and age of patients, individual genetic differences and neuroendocrine abnormalities [8]. APs can induce hyperprolactinemia by blocking dopamine receptors in the nodal-funnel pathway of the brain. One study [9] showed that hyperprolactinemia reduces the body's sensitivity to insulin and leads to higher blood glucose. Simon *et al.* in 2009 showed a relation between the incidence of metabolic side effects and olanzapine or clozapine plasma concentration levels [10]. Yoshida *et al.* generalized the relevant literatures and found dose-dependence of metabolic and cardiovascular disease with APs [11]. In recent years, AIMetD related reviews have been published successively [12-15], which summarized the progress from the perspectives of drug regimen, molecular mechanism, and metabolomics. However, these articles only illustrated the situation from a specific aspect of AIMetD and failed to point out current hotspots and promising topics.

Bibliometric analysis is a statistical method for rapid quantitative analysis and visualization of scientific findings, research hotspots and trends using public literature databases [16]. This method, which is widely used in the medical field [17], allows a systematic analysis of specific research to demonstrate trends over many years and identify scientific frontiers in a short time. Although researches on APs and MetD widely exist worldwide, to our knowledge, there has been no systematic analysis using bibliometrics on this topic. The aim of this study is to fill the gaps in traditional reviews. Since previous studies did not necessarily use the diagnostic criteria of MetS, to include more relevant literatures, this study has extended the concept of metabolic change. Applying bibliometric and visual analysis, the research status and development trend of AIMetD from 2006 to 2021 will be described in detail.

## 2 MATERIALS AND METHODS

### 2.1 Data Acquisition and Search Terms

Web of Science (WoS), previously known as Web of Knowledge, is an online science citation indexing service maintained by Thomson Reuters [18]. Web of Science Core Collection (WoSCC) is one of the most influential databases [19], where the publications included have been rigorously evaluated. All data for this study were obtained from WoSCC. As the database is kept open and a new index is added daily, we performed a retrieval from WoSCC on the date of 24 September 2022 to avoid bias. The search index utilized in WoSCC is standardized to include all relevant publications to ensure the comprehensiveness of data.

Synonyms for antipsychotics and metabolic syndrome were included in the search strategy, as follows: TS = (((metabolic OR metabolism) AND (syndrome OR disorder OR disturbance OR abnormality)) OR (MetS OR MS)) AND TS= (((antipsychotic OR psychotic) AND (drug OR agent OR medication OR medicine)) OR (antipsychotics)). In this study, the inclusive and exclusion criteria were as follows: (I) the timespan ranged from 2006 to 2021, encompassing 15 years in total; (II) only articles and reviews were included, whereas other document types (*e.g*., letters, meeting abstracts, retracted publications, and book chapters) were excluded, (III) no species limitations were set, (IV) the publication language was restricted to English, and (V) duplicate publications were excluded. Export the retrieval results to a plain text format, followed by analysis *via* CiteSpace, VOSviewer and biblioshiny. The data used in the study were obtained from published secondary literature and did not involve any human subjects. Therefore, the requirement for institutional review board approval was waived [20].

### 2.2 Analysis Tools

The h-index of researcher and the impact factor (IF) of the journal are consulted from WoS. The h-index is the number of articles that have been cited at least *h* times each, which assesses the cumulative research output and impact [21]. The IF from Thomson Reuters indicates the impact of journals, referring to the number of citations to a given journal in a specific year [22].

R-biblometrix (4.0.0) (http://www.bibliometrix.org) is a resource package based on R-studio that allows to analyze and visualize the data online. Biblioshiny is an application in R-biblometrix that, when executed, provides a web interface to analyze the imported data. This study uses Biblioshiny to conduct descriptive analysis and create country/region collaboration maps and “keywords+” thematic maps.

CiteSpace (6.1 R3) (http://cluster.cis.drexel.edu/~cchen/citespace/) is a Java-based literature analysis software. Collaborative network analysis of authors, countries/regions and institutions, co-citation analysis of journals and references, and burst detection of keywords and references were performed by CiteSpace. The specific parameters used in CiteSpace were set as follows: time slicing (from 2006 to 2021, years per slice =1), term source (title, abstract, author, keyword, and keywords plus), node type (one option chosen at a time from author, institution, country, keyword, cited reference, cited author, and cited journal), selection criteria (top 50 per slice).

VOSviewer (1.6.18) (https://www.vosviewer.com/download) is used for co-occurrence keyword analysis and generates the network. The parameters for analyzing co-occurrence keywords are set as follows: counting method (fractional counting), minimum number of citations of a source (100). Other parameters are set in default values.

Use IBM SPSS Statistics version 25.0 for statistical analysis. Pearson correlation coefficient (r) was used to evaluate the correlation between the selected continuous variables. *P* < 0.05 was considered statistically significant.

## 3 RESULTS

### 3.1 Analysis of Publication Outputs

**Fig. (1):**
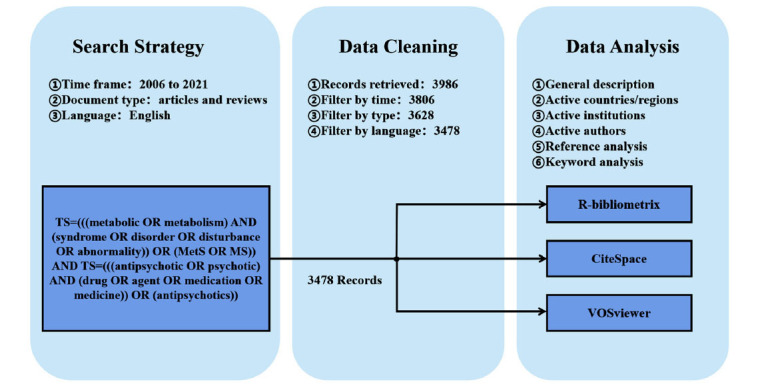
Flowchart for included and excluded literature studies.

**Fig. (2):**
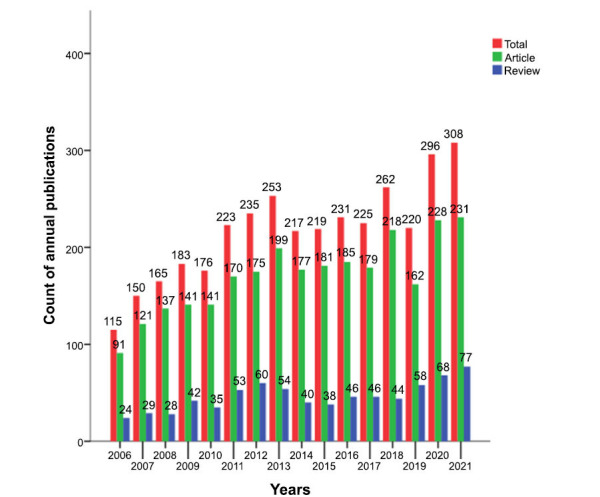
The annual number of publications.

This study retrieved 3478 papers that met the inclusion (Fig. **1**), including 2736 articles and 742 reviews. Over the past 15 years, the trends in AIMetD can be divided into 3 phases (Fig. **2**): The first phase was from 2006 to 2013 when the number of publications produced per year increased steadily; the second phase was from 2014 to 2018, a phase in which the trend showed several consecutive years of highs and lows, with the quantity decreasing to the level of 217-231 per year; the third phase is from 2019 to 2021 when the outputs increase significantly. Overall, it shows a fluctuating upward trend. The annual growth rates of total publications, articles and reviews are 6.79%, 6.41% and 8.08%, respectively. As shown in Fig. (**3**), the cumulative growth rates all showed a fluctuating upward trend, with reviews having the maximal growth rate.

### 3.2 Analysis of Scientific Collaboration Network

**Fig. (5):**
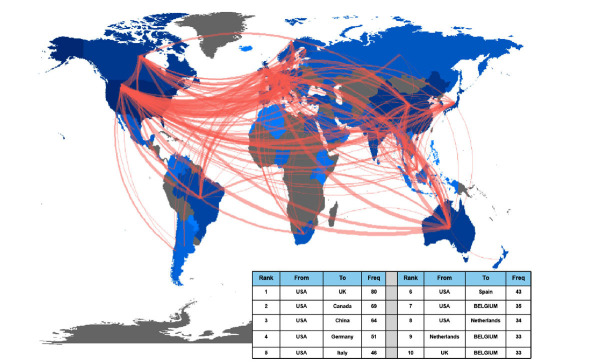
Countries/regions cooperation world map.

Collaboration network analysis helps to assess the current state of research collaboration and to identify key partners. In total, there were 87 countries/regions, 537 scientific institutions and 677 authors contributing to AIMetD between 2006 and 2021. The top 10 items, according to count and centrality, are listed in Table **1**. Pearson’s correlation analysis revealed a significant positive correlation between paper count and centrality, respectively, at country/region, institution and individual level (r = 0.862, r = 0.887 and r = 0.760, *P* < 0.001). Fig. (**4A**-**C**) successively shows the collaboration network between countries/regions, institutions and authors by figurative nodes and lines. A node represents a unit, and the line indicates partnership. The larger the node, the more the paper count. The thicker the line, the more frequent the collaboration. Furthermore, the purple-marked node means that its centrality is over 0.1. In short, the comprehensive evaluation of count and centrality shows that the USA, the UK and Italy are the most influential countries, and the world collaboration map (Fig. **5**) also proves this result. Besides that, it shows that King's College London and Zucker Hills Hospital are the most influential institutions, while Christoph U Correll is the author with the greatest comprehensive influence and highest h-index and is also a researcher at Zucker Hillside Hospital in the USA. According to WoS's records, Christoph U Correll's research area involves psychiatry, neuroscience & neurology, and pharmacy & pharmacology, conforming to the interdisciplinary field of AIMetD. Their powerful research findings have contributed greatly to AIMetD and deserve further attention.

### 3.3 Analysis of Journals and Co-cited Journals

The publications included in this study were issued in 985 journals. Table **2** lists the 10 journals with the most publications and co-citations. Among the retrieved documents, the most productive journal is Schizophrenia Research, followed by Journal of Clinical Psychiatry, Journal of Clinical Psychopharmacology, Frontiers In Psychiatry, and Psychiatry Research, whose volume respectively accounts for 4.17%, 2.56%, 2.18%, 2.07% and 1.93% of the total. The 10 most active journals contributed 20.18% of the total publications. Active journals are mainly located in America, England, and the Netherlands. The IF of these journals is mainly between 4 and 6. Co-citation is one of the important metrics in bibliometrics, which refers to journals that are co-cited by other articles. The top 3 journals ranked according to the number of co-citations are the Journal of Clinical Psychiatry, American Journal of Psychiatry and Schizophrenia Research, which are cited 2251, 2231 and 2202 times, respectively. Taking comprehensive consideration, the Journal of Clinical Psychiatry and Schizophrenia Research are the most preferred journals in the AIMetD study.

### 3.4 Analysis of Co-cited References

Co-cited references mean literatures that are co-cited by different articles. References with high co-citation reflect the scholarly base of a specialized field. Fig. (**6**) shows the clustering diagram of the co-cited references with a total of 67 clusters generated. The nodes represent the cited references, the blocks of different colors stand for different clusters, and the representative literature is labeled next to each cluster. Silhouette value is an index to evaluate the homogeneity of the network, the closer to 1, the higher the homogeneity, when the value > 0.7 can be considered as good tightness of the network [23]. Q-value > 0.3 identified the cluster structure as significant. The silhouette values of the clusters are all > 0.7, and the Q-value is 0.6616, indicating a convincing profile. The 5 largest clusters are “antipsychotic-induced metabolic dysfunction” (cluster # 0), “first-episode schizophrenia” (cluster # 1), “metabolic syndrome” (cluster # 2), “drug-naive population” (cluster # 3) and “systemic review” (cluster # 4), reflecting the important base in this area. The top 10 co-cited references are shown in Table **3** [24-32]. The most co-cited literature was published by Jeffrey A Lieberman with 177 citations and is in cluster #4. The article by Lieberman *et al.* was published in 2005, belonging to the Clinical Antipsychotic Trials of Intervention Effectiveness (CATIE) [24]. One thousand four hundred ninety-three patients with schizophrenia were randomly assigned to the fenadine and AAPs groups to compare the efficacy. AAPs were found to be effective, but the incidence of discontinuation due to side effects was as high as 64% to 82%, with olanzapine having the highest rate and the most common side effects being weight gain and metabolic disturbances. The second most co-cited literature is a review published by Newcomer *et al.* in 2005, summarizing the effects of AAPs on metabolic processes *in vivo* [25]. The review was published early and summarized the research progress at that time, so it is often cited in follow-up work. Therefore, for those who are first experimenting with AIMetD, these 2 articles have the value of quickly finding out the foundation.

### 3.5 Analysis of Co-occurrence Keywords and Thematic Map

Co-occurrence keywords analysis can reflect the co-occurrence relationship of keywords in a series of research and present the key topics in a certain period, which is helpful for researchers to know the progress in time. The co-occurrence keywords network is shown in Fig. (**7**), and the size of the node reflects the co-occurrence frequency of the keywords. The most representative keywords are “schizophrenia”, “metabolic syndrome,” and “double-blind.” The different clusters indicate the most frequently co-occurring keywords in different research aspects. The “schizophrenia” is in the blue cluster, and other keywords within this cluster are “olanzapine”, “clozapine, “induced weight gain” and “insulin-resistance”; “metabolic syndrome” is located in the green cluster with other keywords “weight-gain”, “obesity”, “risk “and “prevalence”; “double-blind” belongs to the red cluster, and other keywords are “bipolar disorder”, “risperidone”, “efficacy” and “aripiprazole”. Inferentially, the blue cluster is related to therapeutic drugs for schizophrenia and their effects on blood glucose and body weight. The green cluster aims to account for the risk of MetD and weight gain, while the red cluster focuses mainly on clinical trials of psychiatric drugs. It can be concluded that the current research mainly involves glucometabolic dysfunction, the risk of MetD and drug clinical trials. Relatively small yellow clusters, including keywords such as “LC-MS/MS”, “tandem mass spectroscopy”, and “validation”, may indicate that detection technologies have a certain position in AIMetD.

By analyzing the thematic map of “Keyword+”, we can further identify the relevant topics in this area and judge their development. Fig. (**8**) shows the topic map of “Keyword+”, with the X-axis indicating the centrality (importance) of the topic and the Y-axis indicating the density (development) of the topic [33]. According to Cobo *et al.* [34], the first quadrant belongs to motor themes, characterized as important and well-developed. The clusters in the first quadrant are “schizophrenia”, which includes the keywords “metabolic syndrome”, “risk”, and “weight-gain”; and the cluster “tandem mass-spectrometry”, which includes the keywords “human plasma”, “metabolites”, and “liquid-chromatography”. The keywords in the cluster “schizophrenia” are consistent with the search topic, signifying that the general direction of the study has been highly developed. In psychiatric treatment, especially schizophrenia, the induction of MetD is more valued in the past. However, the centrality and density of “tandem mass-spectrometry” are relatively low, implying that its importance in AIMetD is boosting. That is to say, analytical technologies such as liquid chromatography and mass spectrometry have good development potential in follow-up research.

### 3.6 Analysis of Burst Detection

The burst keywords in AIMetD from 2006 to 2021 were obtained by Citespace, which can reflect the frontier research themes. Fig. (**9**) shows the top 30 keywords with the strongest burst strength. The strongest keyword is “diabetes mellitus” (strength: 15.41), followed by “major depressive disorder” (strength: 14.28), “meta-analysis” (strength: 14.09), and “schizoaffective disorder” (strength: 11.17). “Diabetes mellitus”, “olanzapine”, “coronary heart disease”, and “haloperidol” emerged in 2006, suggesting that these topics may have received widespread attention before 2006. Other keywords worth noting are “CATIE schizophrenia trial” and “LC-MS/MS”. The burst of “CATIE schizophrenia trial” is strong and has lasted for 5 years. Although the burst of “LC-MS/MS” is relatively weak, it has lasted for a long time, from 2015 to the present. The burst of “expression” and “homeostasis” in 2019 suggests that the newest hotspots may be related to gene or target expression and metabolic homeostasis.

Burst references refer to literature whose number of citations increases with time and can be used to indicate the evolution of topical knowledge in a field. Fig. (**10**) lists the top 30 citations with the strongest burst strength, and the red lines represent the duration of the burst. The earliest 9 citations appeared in 2006, among which the article published by Lieberman *et al.* [24] had the strongest burst, bursting in 2006 and lasting until 2010. It is also the most co-cited article, indicating that this literature is fundamental and powerful in AIMetD. The longest burst duration of references was 5 years [29, 35]. Notably, recent burst citations appeared in 2018 hence, glucose homeostasis [36] and major depressive disorder (MDD) [37] may be new trending knowledge. In general, it is recommended to consult Jeffrey A Lieberman's article in 2006 for the background before conducting the initial AIMetD study. Moreover, it is possible to further explore the alteration process of glucose homeostasis and the bond between medication for MDD and metabolic abnormality.

## 4 DISCUSSION

### 4.1 General Trends

In this study, bibliometric analyses and network visualizations were conducted to characterize the knowledge domains of antipsychotics related to metabolic syndrome. Research contributions, development status and recent frontiers were also identified.

Since 2006, the publication number of AIMetD-related articles has shown a fluctuating upward trend. The most influential countries and institutions are the United States and King's College London. The USA has the largest number of publications, the highest centrality among countries/regions and the strongest collaborations worldwide. The UK is closely behind, with the second-largest number of publications and the second-highest centrality. This high level of development can be explained by their strong economic strength with more funds and assistance in scientific research. Although China ranks fourth in number, its centrality is low. China's low impact is due to its status as a developing country, late start in research, and shortage of international cooperation. The most influential institutions for comprehensive evaluation of number and centrality come from the USA (Zucker Hillside Hospital) and the UK (King's College London). Therefore, the USA and the UK are currently the world leader in the AIMetD domain and have a significant impact on this direction.

### 4.2 Influential Journals and Authors

The top 10 most active journals have published 702 articles, accounting for about 1/5 of the total, which reveals the dispersion of submissions. The probable reason is that AIMetD research involves multiple disciplines (such as psychiatry, pharmacology, neuroscience, *etc*.), and the authors have a variety of journals to choose. In terms of number and co-citations, Journal of Clinical Psychiatry and Schizophrenia Research are the most active journals in this field. After 2021, these two journals also continued to publish high-quality articles about AIMetD. In the Journal of Clinical Psychiatry, Piras *et al.* [38] published a prospective study that evaluated whether there was a dose-dependent effect of risperidone on MetD by analyzing the relationship between the dose and the changes in body weight, blood lipids, blood glucose and blood pressure. Another study aimed to evaluate the correlation between 11 different APs and metabolic side effects through meta-analysis [39]. Schizophrenia Research has also successively published related studies. In a longitudinal study, Segura *et al.* [40] evaluated the effect of polygenic risk score (PRS) on AIMetD in patients with schizophrenia, finding that high-risk patients with depression and cholesterol related PRS had higher blood lipid levels during follow-up. Shymko *et al*. [41] conducted further research on aripiprazole, and found that in patients with early psychosis, compared with oral preparations, long-acting injectable formulations reduce the risk of weight gain. Additionally, the MetD incidence rate of psychiatric inpatients in the United States between 1993 and 2018 was reported [42]. During the widespread use of AAPs, the risk of cardiometabolic abnormality in adult inpatients is increased. Whether there is AAPs exposure or not, the risk of MetD is higher than that of other populations.

The most prolific and central author is Christoph U Correll, who is also a researcher at Zucker Hillside Hospital in the USA, with influential contributions. One of the previous studies is a strong burst citation, which described the risk of cardiometabolic disorder caused by the first use of AAPs in children and adolescents [31]. Additionally, Christoph U Correll’s other 3 articles are identified as strong busrt citations. The article in 2014, part of the National Institute of Mental Health-funded Recovery After an Initial Schizophrenia Episode-Early Treatment Program (RAISE-ETP), analyzed risk factors for the development of cardiometabolic abnormality in patients with first-episode schizophrenia spectrum disorders [43]. The review in 2015 summarized the effects of antipsychotics, antidepressants and mood stabilizers on patients and believed that these drugs were associated with a high risk of hyperlipidemia, diabetes, cardiovascular disease and kidney disease [35]. A recent burst citation is issued by Correll *et al*. [37], a large meta-analysis evaluating the incidence and mortality of cardiovascular disease (CVD) in severely psychotic patients and indicating that APs are one of the risk factors for CVD. Notably, Christoph U Correll published a new finding in 2022 [44], a 24-week phase 3 double-blind clinical trial, which evaluated the effect of olanzapine monotherapy and the combination of olanzapine and samidofen on cardiovascular metabolism. It can be concluded that Christoph U Correll has been committed to AIMetD research, particularly in the field of cardiovascular metabolism. Several researches reported that APs can increase CVD-related mortality [27, 35] and the risk of cardiometabolic abnormality [45, 46]. Carefully weighing the therapeutic effects of different APs against the risk of metabolic side effects and individualizing treatment for different patients are key to producing favorable effects. Those prove that cardiometabolic disorder is one of the emphases of AIMetD, deserving more attention and even seeking cooperation from influential researchers.

### 4.3 Outlook

Our co-occurrence networks, thematic maps and burst detection shed light on the outcome that the current hotspots and future directions in the association of APs with the metabolic disorder may be divided into the following branches: promising research method and resource, potential biological mechanism, and psychiatric comorbidity.

### 4.4 Promising Research Method and Resource

In the co-occurrence network, the yellow cluster is related to the analysis methods in AIMetD, where “LC-MS/MS” has links with many drugs and biological matrices. In the thematic map, the clustering keywords in the first quadrant include “tandem mass-spectrometry”, while”LC-MS/MS” burst in 2015 and last until recently. This series of results show that LC-MS/MS has been applied more and more frequently in AIMetD and may be a promising method in the future. LC-MS/MS is an advanced analytical technology which is widely used in many medical fields [47, 48]. Moreover, because LC-MS/MS is extremely sensitive, it can quantify substances at a very low level and simultaneously determine a variety of compounds in a large sample set, providing results with high accuracy and precision [49]. This technology has been used in the therapeutic drug monitoring [50, 51] and forensic analysis [52, 53] of APs. LC-MS/MS quantitative analysis of APs is mainly based on the reverse phase chromatography of hydrophobic interaction, which uses isocratic elution or gradient elution. The commonly used mobile phase is acetonitrile or a mixture of methanol and water in different proportions. In recent studies, LC-MS/MS has been mostly applied to determine *in vivo* metabolites to evaluate metabolic alterations caused by antipsychotics. Lipids are widely present in the brain and play an important role in the regulation of membrane composition, energy metabolism, neural signaling and even the occurrence of schizophrenia [54]. Alterations in lipid homeostasis may be caused by APs promoting membrane phospholipid breakdown and inducing inflammatory responses [55, 56]. Valéria de Almeida *et al.* [57] investigated the relationship between APPs-related lipid alterations and the response to treatments. Applying LC-MS/MS to obtain lipid profiles in schizophrenic patients before and after receiving APPs. Comparing the profiles, it was found that olanzapine and risperidone could affect many kinds of lipids, while quetiapine had less effect. Based on previous studies [58-61], progesterone receptor membrane component 1 (PGRMC1) is associated with lipid and glucose homeostasis degradation induced by AAPs. Cao *et al.* [62] studied the effect of the PGRMC1 signal on liver glucose metabolism by giving clozapine to male mice for 4 weeks. LC-MS/MS is used to determine the concentration of progesterone (PROG) in the plasma, liver and adrenal gland of mice. The binding affinity between PROG and PGRMC1 is the strongest [63]. Measuring the concentration of PROG is helpful to evaluate the effect of clozapine on hormone secretion. Based on the results of LC-MS/MS, Western blotting and other methods, it explained that clozapine in the liver caused glucose metabolism disorder by inhibiting signal transduction. By simulating the knockout and inhibition of PGRMC1, its overexpression could slightly reduce the glucose imbalance caused by clozapine. The high-quality data obtained by LC-MS/MS have greatly promoted the progress of AIMetD.

Reference resources are a part of research, and the premium results are the foundation for further research. The NIMH-funded CATIE Study was a nationwide public health-focused clinical trial that compared the effectiveness of older and newer APs. CATIE compares AAPs with representative first-generation APs and provides rich and reliable information about the advantages and disadvantages of the two drugs. The most frequently cited study is Jeffrey A Lieberman’s research, which also is a part of CATIE [24]. While comparing the efficacy difference between perphenazine and some AAPs, it was found that olanzapine and clozapine are prone to metabolic side effects and lead to discontinuation of the medication. According to the burst citation, this article is also the citation with the earliest and strongest burst, which reflects that this research may be the academic basis of the early stage. Since then, Jeffrey A Lieberman has successively published other studies based on CATIE [64, 65] to evaluate the clinical efficacy of various AAPs. From here, we see that Jeffrey A Lieberman had a great influence at that period. However, in recent years, Jeffrey A Lieberman's research has focused more on the pathogenesis of mental disorders [66], and less on AIMetD. To gain a deeper understanding of this area, we can pay attention to CATIE research.

### 4.5 Potential Biological Mechanisms

After treatment with APs, the expression of genes or targets changes in patients with psychosis, which may be a potential mechanism that exacerbates the onset of AIMetD. Sainz *et al.* [67] sequenced the total mRNA of schizophrenic patients before and after treatment with APs for 3 months and found that there was a significant difference in the expression of 331 genes in male patients with weight gain after treatment, while 119 genes were found in female. According to the GeneRIF database, most of these genes are related to “obesity” and “BMI.” Similarly, in another study, total mRNA sequencing was carried out 3 months before and after the treatment and it was found that the expression of 115 genes in the weight gain group had significant differences. Further comparison with the database showed that the results were similar to the above studies [68]. Further sequencing of the whole genome of European and African-American schizophrenic patients treated with AAPs found that CIDEA and DGKB gene mutations were risk factors for AIMetD in cross-racial populations, and the high induction might be related to G6PD and IRS1 gene pathways. These studies used gene sequencing to analyze the correlation between AIMetS and gene expression changes, providing new ideas and references for the research on differentially expressed genes.

The imbalance of glucose and lipid homeostasis plays an important role in the process of inducing AIMetD and may involve different targets of multiple organs and tissues. The maintenance of this energy homeostasis mainly depends on pancreatic β-cells, liver, skeletal muscle and adipose tissue, which coordinate with each other and are regulated by the hypothalamus [13]. Many G protein-coupled receptors (GPCRs) are distributed in these tissues and organs, and the main ones that can finely regulate metabolic homeostasis include H_1_ receptors, D_2_ receptors, α_2_ receptors, M_3_ receptors, 5-HT_2A_ and 5-HT_2C_ receptors, *etc*. Most of the AAPs have targeted these GPCRs. For example, glucose transporter protein 4-mediated glycogen synthesis and glucose uptake in skeletal muscle require the involvement of H_1_ receptors and 5-HT_2A_ receptors [69-70]. AAPs can disrupt metabolic activity by interfering with the serotoninization and phosphorylation processes in pathway activation [71, 72] through antagonistic effects. The binding affinity of AAPs to M_3_ receptors has been demonstrated to be one of the predictors of AIMetD [73]. Drugs with high M_3_ affinity, such as clozapine and olanzapine, can act on β-cells or interfere with cholinergic pathways in the hypothalamus, directly or indirectly impairing islet secretory function [74]. D_2_ receptors are closely linked to the brain reward system, and the agonism or antagonism of D_2_ receptors can both alter appetite [75]. AAPs antagonize D_2_ and D_3_ receptors in the arcuate nucleus of the hypothalamus, increasing hunger and food intake, which finally leads to weight gain. The imbalance of energy homeostasis is a key process to AIMetD, but there are still many blanks or ambiguities that still need further investigation.

### 4.6 Psychiatric Comorbidity

Recent burst citations and keywords indicate that MDD is a new hotspot in the AIMetD, which may be comorbidity existing in schizophrenic patients. Similarly, MDD is also associated with metabolic disorders. A proteomic analysis found that insulin and leptin in the plasma of patients with MDD or schizophrenia increase [76], illustrating that MDD patients already have metabolic abnormalities before medical treatment. In other correlation studies [77, 78], suicide attempts in first-episode MDD patients were significantly correlated with high blood glucose and lipid level, explaining that MDD patients already have metabolic disorders before medication and glucose and lipid may be a potential biomarker for first-episode MDD suicide risk. Lately, the combination of APs and antidepressants is the new method to solve treatment-resistant MDD. A meta-analysis evaluated the efficacy and tolerability of combination treatments for major depression [79], when antidepressants combine aripiprazole, risperidone, quetiapine or other AAPs, the efficacy is better than monotherapy. Nonetheless, given the risk of AIMetD, attention should be paid to the risk of worsening metabolic disorders in MDD. MDD and its medication therapy-induced metabolic syndrome may be a new hotspot.

## 5 PROSPECT OF INTESTINAL SYSTEM

Although the results of this study do not point to the function of the intestinal system and microbes, multiple studies [80, 81] have shown that microbiota-gut-brain axis is associated with central nervous system (CNS) diseases. Intestinal microbiota plays an important role in stabilizing the internal environment, by decomposition, to generate various substances and regulate metabolism and immune processes. Previous study [82] found that there is an increase in facultative anaerobic bacteria and functional changes of fatty acid synthesis, tryptophan metabolism, and the synthesis or degradation of neurotransmitters in schizophrenic patients. It is noteworthy that 95% of serotonin produced by intestinal system, and then it is transported to the entire body *via* platelets and enters CNS with the help of serotonin transporters on the blood-brain barrier [83]. Inflammatory reactions can disrupt the microbiota to reduce the conversion of tryptophan to serotonin, resulting in abnormal substance metabolism and mental diseases. Based on other studies, taking clozapine and schizophrenia might reduce the richness of gut microbiota in female [84], while the AAPs-induced weight gain might occur *via* alteration of gut microbiota [85]. In sum, both the occurrence of psychosis and AIMetD seem to be related to changes in gut microbiota, hence we also suggest a close attention to microbiota-gut-brain axis.

## 6 LIMITATION

There are some noteworthy limitations of this study. Firstly, we only analyzed publications in the WoSCC, where the language was English. Moreover, the inclusion of articles that may have authors with the same name or terms that mean the same thing but express it differently can affect the results. Despite these limitations, given enough included literatures, we believe that the results of this study can still be used to describe the general situation and trends in AIMetD research.

## CONCLUSION

This study analyzed the last 15 years of AIMetD research by bibliometric and visual analysis. Since 2006, the number of articles published has been in a fluctuating growth trend. In general, research in this area has strong scientific collaboration between countries/regions. The most influential countries, scientific institutions and authors are the USA, King's College London and Christoph U Correll, respectively. Cardiovascular metabolism and CATIE study may be one of the hotspots. Burst detection indicates “LC-MS/MS”, “major depressive disorder”, “expression”, “homeostasis” are the frontiers of AIMetD. Therefore, our timely review and analysis of the hotspots and research trends may promote the development of this field.

## Figures and Tables

**Fig. (3):**
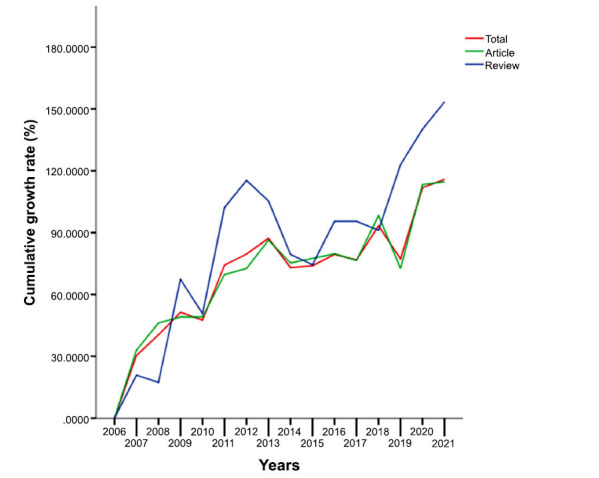
The cumulative growth rate for publications.

**Fig. (4):**
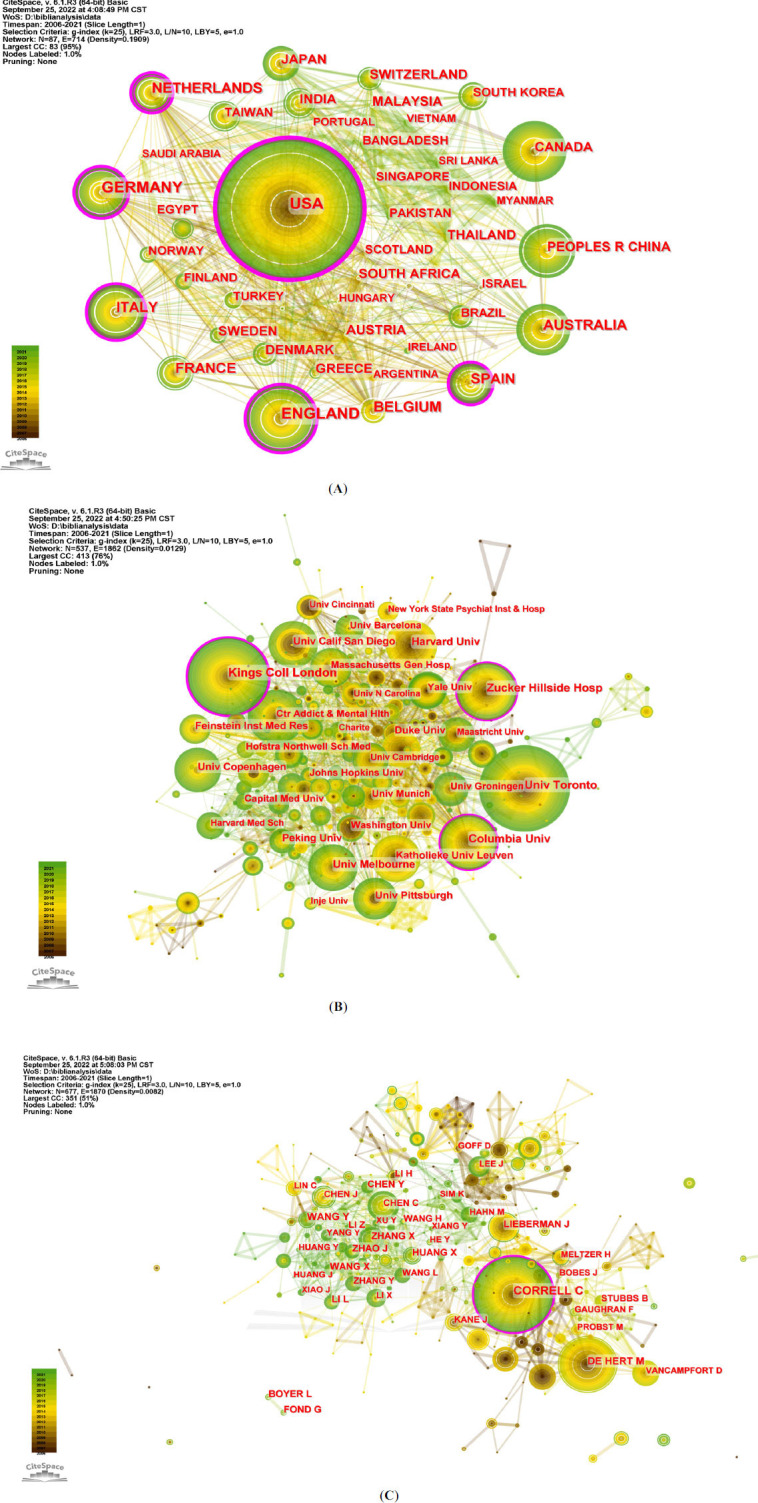
(**A**) Scientific collaboration networks among countries/regions. (**B**) Scientific collaboration networks among institutions. (**C**) Scientific collaboration networks among authors.

**Fig. (6):**
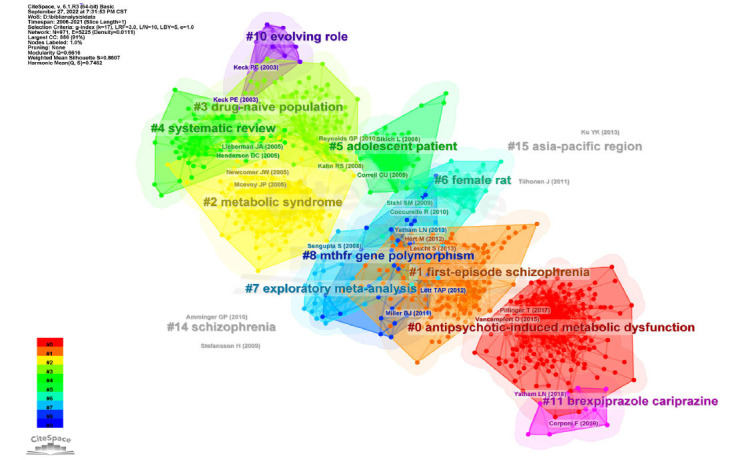
Cluster visualization of the reference co-citation map.

**Fig. (7):**
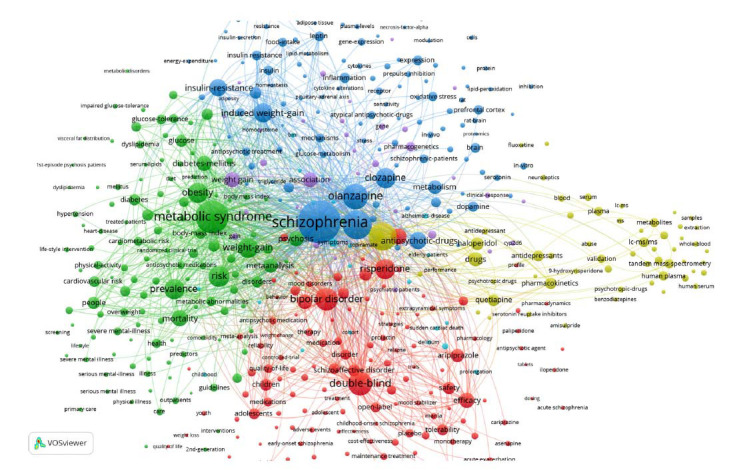
Co-occurring keyword network.

**Fig. (8):**
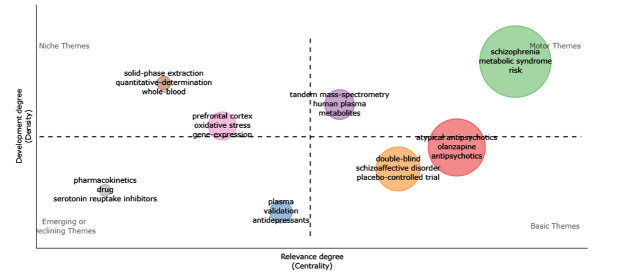
“Keyword+” thematic map.

**Fig. (9):**
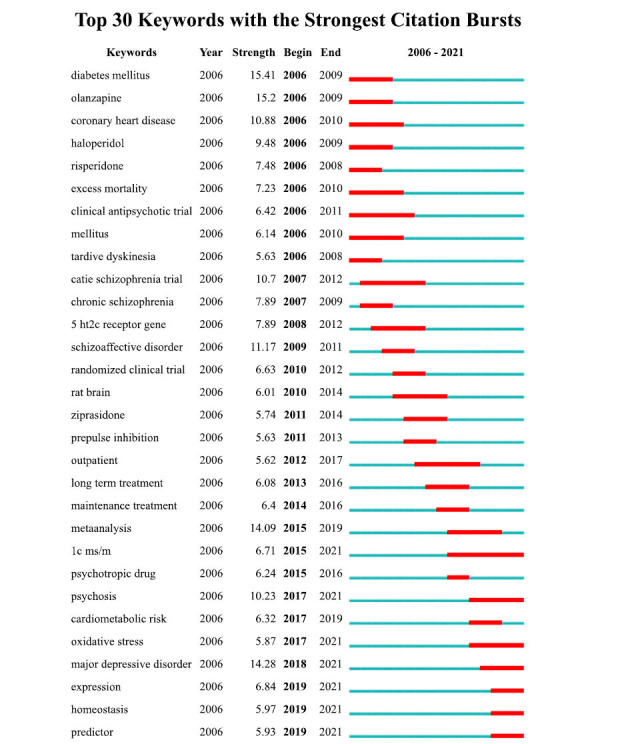
References with the strongest burst in publications from 2006 to 2021 on AIMetD.

**Fig. (10):**
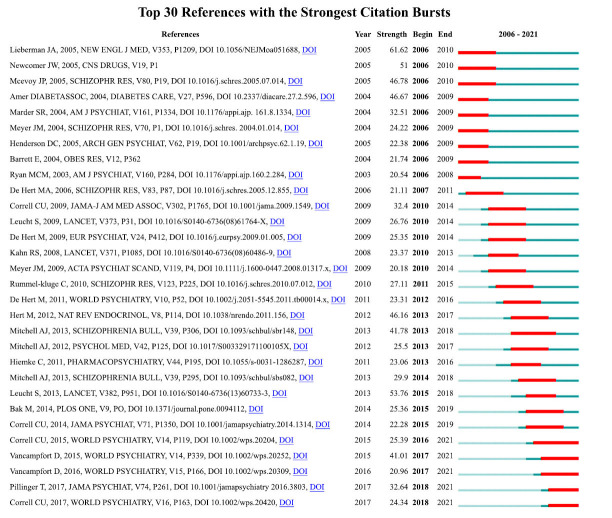
Keywords with the strongest burst in publications from 2006 to 2021 on AIMetD.

**Table 1:** The top 10 countries/regions, institutions, and authors in terms of publications and centrality.

**Items**	**Publications**			**Centrality**		
	**Ranking**	**Name**	**Number**	**Ranking**	**Name**	**Value**
Country/ Region	1	USA	1115	1	USA	0.27
	2	England	314	2	England	0.18
	3	Canada	281	3	Germany	0.15
	4	Peoples R China	276	4	Netherlands	0.14
	5	Italy	237	5	Italy	0.12
	6	Germany	219	6	Spain	0.10
	7	Australia	216	7	Canada	0.09
	8	Spain	173	8	Australia	0.08
	9	Netherlands	149	9	Belgium	0.08
	10	Japan	117	10	France	0.08
Institution	1	University of Toronto	112	1	King's College London	0.24
	2	King's College London	92	2	Zucker Hillside Hospital	0.17
	3	Zucker Hillside Hospital	63	3	Columbia University	0.11
	4	Harvard University	59	4	University of Toronto	0.09
	5	Columbia University	56	5	University of Melbourne	0.09
	6	The Centre for Addiction and Mental Health	54	6	Institut National de la Sante et de la Recherche Medicale	0.08
	7	Katholieke University of Leuven	50	7	Harvard University	0.06
	8	University of California San Diego	48	8	Katholieke University of Leuven	0.06
	9	University of British Columbia	45	9	University of Pittsburgh	0.06
	10	University of Copenhagen	44	10	University of Groningen	0.06
Author	1	Christoph U Correll	92	1	Christoph U Correll	0.24
	2	Marc De Hert	68	2	Chiao-Chicy Chen	0.08
	3	Chiao-Chicy Chen	29	3	Jeffrey A Lieberman	0.05
	4	Jeffrey A Lieberman	26	4	Xin Li	0.05
	5	Jiezhong Chen	26	5	Y He	0.05
	6	Xianhua Zhang	24	6	Jimmy Lee	0.04
	7	Davy Vancampfort	24	7	Margaret K Hahn	0.04
	8	Xin Li	23	8	Hongliang Li	0.04
	9	Sung-Wan Kim	23	9	Hsuan Chi Wang	0.04
	10	Jimmy Lee	22	10	René Kahn	0.04

**Table 2:** The top 10 journals and co-cited journals for atypical antipsychotics-induced metabolic disorder.

**Items**	**Ranking**	**Name**	**Country**	**Counts**	**IF(2021)**
Journal	1	Schizophrenia Research	Netherlands	145	4.662
	2	Journal of Clinical Psychiatry	USA	89	5.906
	3	Journal of Clinical Psychopharmacology	USA	76	3.118
	4	Frontiers in Psychiatry	USA	72	5.435
	5	Psychiatry Research	Netherlands	67	11.225
	6	Neuropsychiatric Disease and Treatment	New Zealand	60	2.989
	7	Progress In Neuro-Psychopharmacology & Biological Psychiatry	England	59	5.201
	8	Bmc Psychiatry	England	47	4.144
	9	Journal of Psychopharmacology	England	44	4.562
	10	Psychopharmacology	Germany	43	4.415
Co-cited Journal	1	Journal of Clinical Psychiatry	USA	2251	5.906
	2	American Journal of Psychiatry	USA	2231	19.242
	3	Schizophrenia Research	Netherlands	2202	4.662
	4	Archives of General Psychiatry	USA	1564	-
	5	Journal of Clinical Psychopharmacology	USA	1506	3.118
	6	British Journal of Psychiatry	England	1469	10.671
	7	Schizophrenia Bulletin	USA	1453	7.348
	8	Lancet	England	1280	202.731
	9	Neuropsychopharmacology	England	1225	8.294
	10	Acta Psychiatrica Scandinavica	England	1179	7.734

**Table 3:** The top10 co-cited references related to atypical antipsychotics-induced metabolic disorder.

**Rank**	**Cited by**	**Authors**	**Title**	**Source**	**Type**	**In Cluster**
1	177	Jeffrey A Lieberman [24]	Effectiveness of antipsychotic drugs in patients with chronic schizophrenia	New England Journal of Medicine	Article	#4
2	147	John W Newcomer [25]	Second-generation (atypical) antipsychotics and metabolic effects: a comprehensive literature review	CNS Drugs	Review	#2
3	135	Joseph P McEvoy [26]	Prevalence of the metabolic syndrome in patients with schizophrenia: baseline results from the Clinical Antipsychotic Trials of Intervention Effectiveness (CATIE) schizophrenia trial and comparison with national estimates from NHANES III	Schizophrenia Research	Article	#2
4	121	Stefan Leucht [4]	Comparative efficacy and tolerability of 15 antipsychotic drugs in schizophrenia: a multiple-treatments meta-analysis	Lancet	Article	#1
5	119	Marc De Hert [27]	Metabolic and cardiovascular adverse effects associated with antipsychotic drugs	Nature Reviews Endocrinology	Review	#1
6	113	American Diabetes Association [28]	Consensus development conference on antipsychotic drugs and obesity and diabetes	Diabetes Care	Review	#2
7	109	Alex J Mitchell [29]	Prevalence of Metabolic Syndrome and Metabolic Abnormalities in Schizophrenia and Related Disorders-A Systematic Review and Meta-Analysis	Schizophrenia Bulletin	Review	#1
8	90	Davy Vancampfort [30]	Risk of metabolic syndrome and its components in people with schizophrenia and related psychotic disorders, bipolar disorder and major depressive disorder: a systematic review and meta-analysis	World Psychiatry	Review	#0
9	88	Christoph U Correll [31]	Cardiometabolic risk of second-generation antipsychotic medications during first-time use in children and adolescents	Jama-Journal of The American Medical Association	Article	#5
10	86	Stefan Leucht [32]	Second-generation *versus* first-generation antipsychotic drugs for schizophrenia: a meta-analysis	Lancet	Article	#2

## Data Availability

The raw data were retrieved based on specific terms in Web of Science Core Collection. The data and materials supporting the conclusions of this article will be made available by the authors without undue reservation.

## LIST OF ABBREVIATIONS

AAPs: Atypical Antipsychotics
AIMetD: Antipsychotic-induced Metabolic Disorder
APs: Antipsychotics
5-HT: 5-hydroxytryptamine
MetS: Metabolic Syndrome
TAPs: Typical Antipsychotics
